# P-1983. Blood Metagenomic Next-Generation Sequencing Outperforms Bronchoalveolar Lavage Fluid in Diagnosing and Prognosticating Pneumocystis jirovecii Pneumonia: A Comprehensive Microbial Landscape Analysis

**DOI:** 10.1093/ofid/ofaf695.2150

**Published:** 2026-01-11

**Authors:** Yuhui Chen, Meng Li, Xinai Gan, Yutong Wang, Yang Yang, Min Huang, Qitong Wang, Pazilaiti Tuohuti, Ting Niu, Yongzhao Zhou

**Affiliations:** Department of Hematology, Institute of Hematology, West China Hospital, Sichuan University, Chengdu, Sichuan, China (People's Republic); West China Hospital, Sichuan University, Chengdu, Sichuan, China; West China Hospital, Sichuan University, Chengdu, Sichuan, China; West China Hospital, Sichuan University, Chengdu, Sichuan, China; West China Hospital, Sichuan University, Chengdu, Sichuan, China; West China Hospital, Sichuan University, Chengdu, Sichuan, China; West China Hospital, Sichuan University, Chengdu, Sichuan, China; West China Hospital, Sichuan University, Chengdu, Sichuan, China; West China Hospital, Sichuan University, Chengdu, Sichuan, China; West China Hospital, Sichuan University, Chengdu, Sichuan, China

## Abstract

**Background:**

*Pneumocystis jirovecii* pneumonia (PJP) remains a significant challenge in immunocompromised patients. This study aimed to evaluate the diagnostic performance of metagenomic next-generation sequencing (mNGS) of blood and bronchoalveolar lavage fluid (BALF) samples for PJP diagnosis, characterize associated microbial communities, assess the consistency between BALF and blood microbiota profiles, and explore the prognostic value of *P. jirovecii* detection by mNGS in both sample types.

**Figure:**
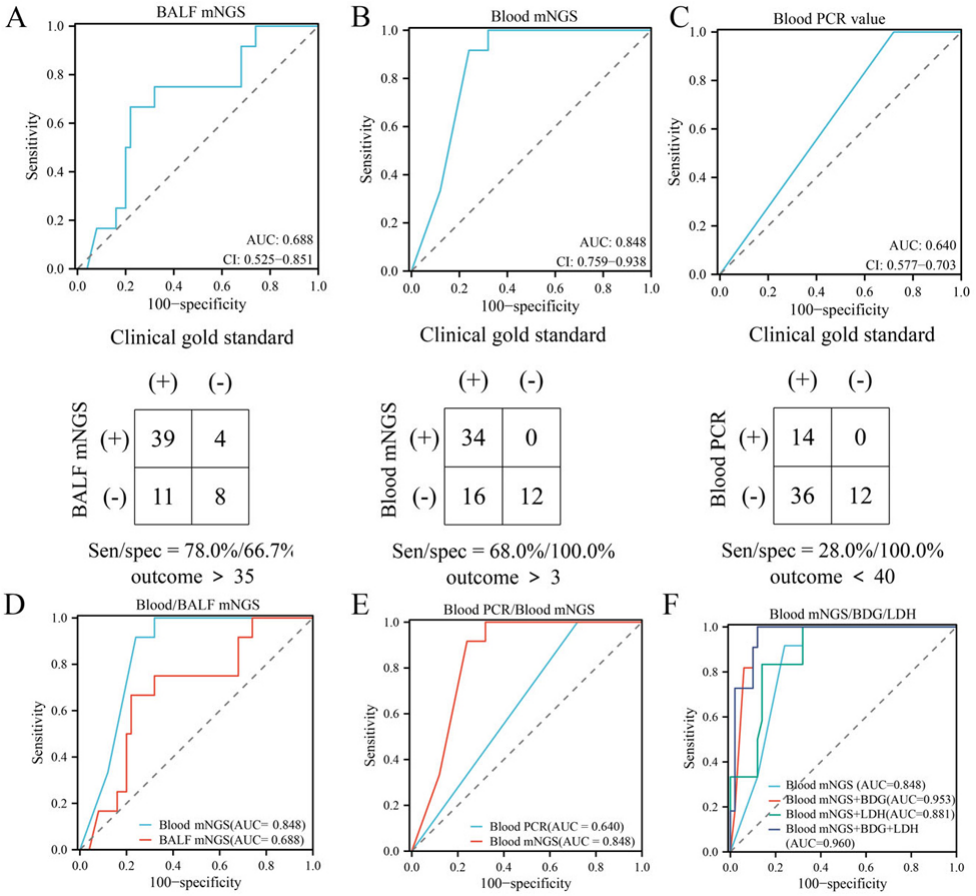
Diagnostic Performance of mNGS and PCR for Pneumocystis jirovecii Pneumonia. (A) ROC curve for BALF mNGS. (B) ROC curve for blood mNGS. (C) ROC curve for blood PCR. (D) Comparison of ROC curves for BALF and blood mNGS. (E) Comparison of ROC curves for blood mNGS and blood PCR. (F) ROC curves for blood mNGS combined with serological indicators.

**Figure:**
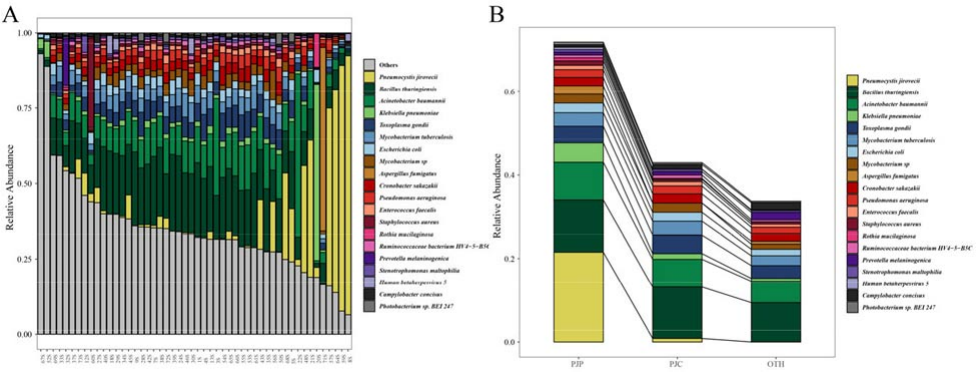
Comparison of Microbial Communities Among PJP, PJC, and OTH Groups. (A) Heatmap showing the top 20 most abundant species detected in BALF specimens across PJP, PJC, and OTH groups. (B) Relative abundance of the top 20 species in each group.

**Methods:**

We retrospectively analyzed 73 patients with suspected PJP who underwent both mNGS of BALF and blood. Patients were categorized as PJP (n=50), *P. jirovecii* colonization (PJC, n=12), or other pneumonia (OTH, n=11). Diagnostic performance of mNGS was compared with conventional blood PCR and serological markers. Microbial community analysis was performed using bioinformatics tools. We assessed the consistency between BALF and blood microbiota profiles in PJP patients and evaluated the association between *P. jirovecii* detection by mNGS and patient outcomes.

**Figure:**
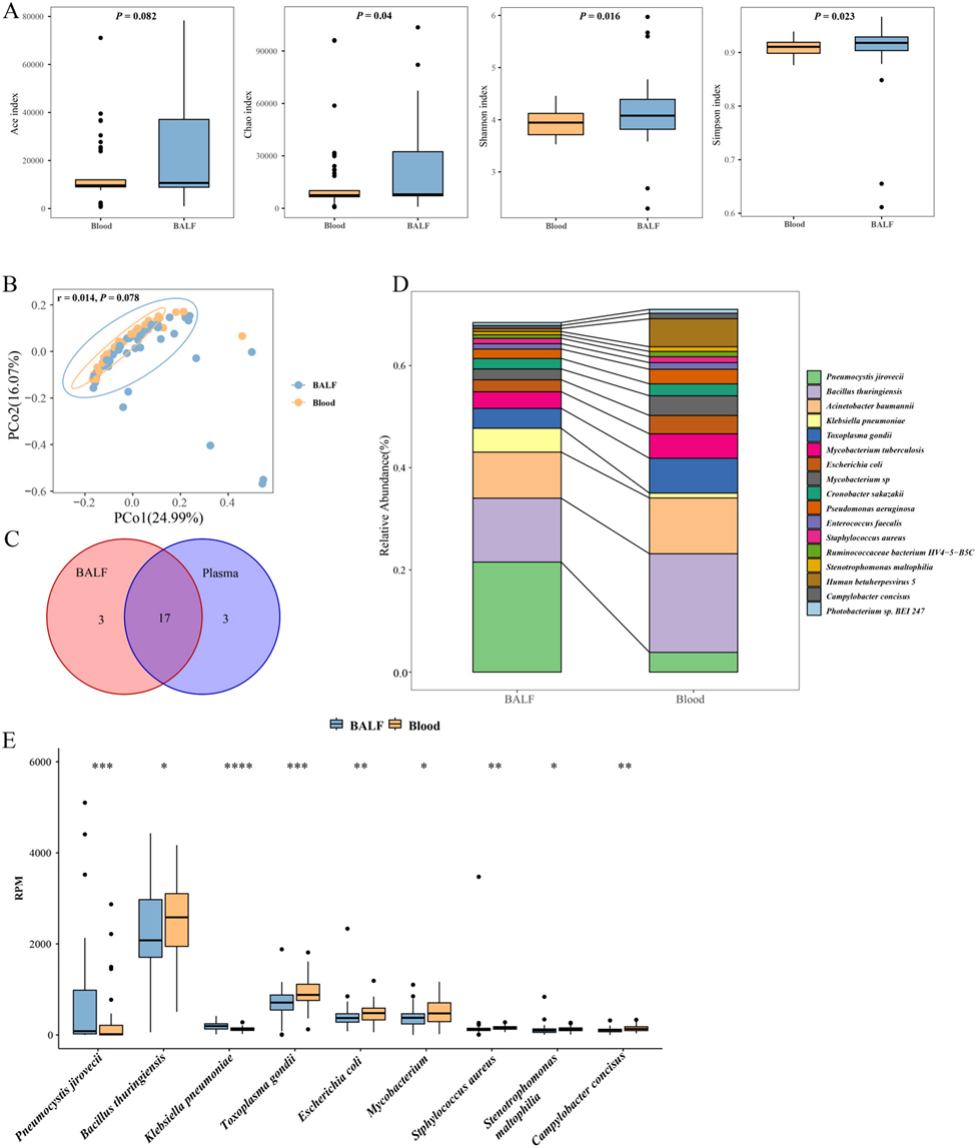
Comparison of Microbial Communities in BALF and Blood Samples from PJP Patients. (A) Alpha diversity analysis at the species level, including Ace, Chao, Shannon, and Simpson indices. (B) Beta diversity analysis at the species level using Bray-Curtis dissimilarity, represented by Principal Coordinate Analysis (PCoA). (C) Venn diagram showing the top 20 microbial species in BALF and blood samples. (D) Relative abundance bar chart of 17 shared microbial species between BALF and blood samples. (E) Box plots of 9 shared microbial species with significantly different abundances between BALF and blood samples. PCoA: Principal Coordinate Analysis.

**Results:**

mNGS of blood demonstrated superior diagnostic performance (AUC 0.848, cutoff >3 RPM) compared to mNGS of BALF (AUC 0.688, cutoff > 35 RPM) and blood PCR (AUC 0.64, Ct < 40). Combining mNGS of blood with serological markers (BDG and LDH) yielded the highest diagnostic accuracy (AUC 0.96). Microbial diversity was comparable across groups, but PJP patients showed higher abundances of various species. BALF and blood samples from PJP patients exhibited consistent microbial compositions with notable differences in species abundance. mNGS of blood showed potential prognostic value, with higher *P. jirovecii* RPM values associated with increased mortality risk (*P* = 0.03).

**Conclusion:**

mNGS of blood, especially when combined with serological markers, offers a promising non-invasive approach for PJP diagnosis. The study provides insights into the microbial landscape of PJP and highlights the potential prognostic value of mNGS of blood, which may improve patient management and outcomes.

**Disclosures:**

All Authors: No reported disclosures

